# In Vitro Activity of Amphotericin B in Combination with Colistin against Fungi Responsible for Invasive Infections

**DOI:** 10.3390/jof8020115

**Published:** 2022-01-26

**Authors:** Patrick Schwarz, Ilya Nikolskiy, Anne-Laure Bidaud, Frank Sommer, Gert Bange, Eric Dannaoui

**Affiliations:** 1 Department of Internal Medicine, Respiratory and Critical Care Medicine, University Hospital Marburg, 35033 Marburg, Germany; 2 Center for Invasive Mycoses and Antifungals, Faculty of Medicine, Philipps University Marburg, 35043 Marburg, Germany; ilian@students.uni-marburg.de; 3 Center for Synthetic Microbiology (SYNMIKRO), Department of Chemistry, Philipps University Marburg, 35043 Marburg, Germany; gert.bange@synmikro.uni-marburg.de; 4 Unité de Parasitologie-Mycologie, Hôpital Européen Georges-Pompidou, 75015 Paris, France; anne-laure.bidaud@aphp.fr (A.-L.B.); eric.dannaoui@aphp.fr (E.D.); 5 Department of Microbiology, University Hospital Marburg, 35032 Marburg, Germany; frank.sommer@med.uni-marburg.de; 6 Max Planck Institute for Terrestrial Microbiology, 35043 Marburg, Germany; 7 Dynamyc Research Group (EA 7380), Faculté de Médecine de Créteil, Université Paris-Est-Créteil-Val-de-Marne, 94010 Créteil, France; 8 Faculté de Médecine, Université de Paris, 75006 Paris, France

**Keywords:** amphotericin B, antifungal combination, *Aspergillus*, *Candida*, colistin, in vitro, colistin, EUCAST, *Rhizopus*

## Abstract

The in vitro interaction of amphotericin B in combination with colistin was evaluated against a total of 86 strains comprising of 47 *Candida* species (10 *Candida albicans*, 15 *Candida auris*, five *Candida glabrata*, three *Candida kefyr*, five *Candida krusei*, four *Candida parapsilosis* and five *Candida tropicalis*), 29 *Aspergillus* species (five *Aspergillus flavus*, 10 *Aspergillus fumigatus*, four *Aspergillus nidulans*, five *Aspergillus niger*, and five *Aspergillus terreus*), and 10 *Rhizopus* species (seven *Rhizopus arrhizus*, one *Rhizopus delemar* and two *Rhizopus microsporus*) strains. For the determination of the interaction, a microdilution checkerboard technique based on the European Committee on Antimicrobial Susceptibility Testing (EUCAST) reference method for antifungal susceptibility testing was used. Results of the checkerboard technique were evaluated by the fractional inhibitory concentration index (FICI) based on the Loewe additivity model for all isolates. Different inhibition endpoints were used to capture both the interaction at MIC and sub-MIC levels. Additionally, checkerboard technique results for *Candida* species were evaluated by response surface analysis based on the Bliss independence model. Against common *Candida* species, the combination was synergistic for 75% of the strains by FICI and for 66% of the strains by response surface analysis. For *C. tropicalis,* the interaction was antagonistic for three isolates by FICI, but antagonism was not confirmed by response surface analysis. Interestingly, synergistic and antagonistic FICIs were simultaneously present on checkboard microplates of all three strains. Against *C. auris* the combination was synergistic for 73% of the strains by response surface analysis and for 33% of the strains by FICI. This discrepancy could be related to the insensitivity of the FICI to detect weak interactions. Interaction for all other strains was indifferent. For *Aspergillus* and *Rhizopus* species combination exhibited only indifferent interactions against all tested strains.

## 1. Introduction

Fungal infections are a leading cause of mortality, especially in immunocompromised patients. Both yeast and filamentous invasive fungal infections are associated with poor outcomes and high mortality rates. In Europe, aspergillosis and mucormycosis are the two most frequent filamentous fungal infections with mortality rates in immunocompromised patients of about 60 and 53%, respectively [1,2,3]. Since the outbreak of the COVID-19 pandemic, not only immunocompromised patients are at risk. COVID-19 associated pulmonary aspergillosis may affect patients’ acute respiratory distress syndrome due to severe COVID-19 infection with an overall incidence during the first wave of 15% in France [4], and 18% in Germany [5]. Day-90 intensive care unit mortality rate was 71% for patients with COVID-19 associated pulmonary aspergillosis versus 43% for patients without [6]. Not only aspergillosis is a problem in severely ill COVID-19 patients, but also mucormycosis, and candidiasis [7]. The predominant form of COVID-19 associated mucormycosis is the rhino-orbital form. In India and in the rest of the world mortality rates of 37% and 62% have been reported, respectively. This discrepancy is most likely related to the fact that in the rest of the world significantly higher numbers of pulmonary and disseminated forms are seen than in India [8]. Severe COVID-19 infection is also a risk factor for invasive candidiasis [9], and outbreaks of multidrug-resistant *Candida auris* have been reported [10]. Although COVID-unassociated mortality rates of invasive candidiasis due to common *Candida* species or *C. auris* of more than 35% are already high [11,12], COVID-associated mortality rates of about 45% for common *Candida* species [13], and 60% for *C. auris* are even higher [14].

In Europe, first-line therapy for aspergillosis is voriconazole or isavuconazole [15], but increasing azole-resistance in *Aspergillus fumigatus* may complicate the treatment [16]. First-line therapy for mucormycosis is liposomal amphotericin B, or isavuconazole when amphotericin B is not possible [17], but amphotericin B therapy is associated with nephrotoxicity. *Candida* infections are preferably treated with echinocandins, or azoles as step-down therapy [18], but high rates of resistance in *Candida glabrata* [19], and *C. auris* have been reported [20]. The above-mentioned high mortality rates highlight that despite advances in the development of new antifungals in the last decades, these drugs still lack efficacy used in monotherapy. The use of combination therapies is a well-known strategy in oncology, to manage problems in efficacy, resistance and toxicity [21]. Moreover, antifungal combinations have also been implemented in fungal infections to overcome resistance, to increase efficacy yielding to synergy, and to reduce toxicity by decreasing dosages [22]. Due to the rarity of the diseases, only two prospective studies evaluated combination therapies for aspergillosis and mucormycosis. A combination of two antifungal drugs, voriconazole and anidulafungin, showed only indifference for the treatment of aspergillosis [23], while the combination of an antifungal with an iron chelator, liposomal amphotericin B and deferasirox even exhibited antagonism for the treatment of mucormycosis [24]. Combination therapy for the treatment of candidiasis was evaluated by only one large study, the combination of amphotericin B with fluconazole was not superior to fluconazole monotherapy [25]. Compared to clinical trials, in vitro studies are easy to conduct, giving the possibility to explore a large number of different combinations, even if there is no immediate translation to the patient. In vitro combinations on two antifungal drugs against *Aspergillus* and Mucorales species showed divers outcomes, but no strong synergistic interactions could be identified [26,27]. Against *Candida*, in vitro combinations showed promising results, but did not lead to an application in uncomplicated candidiasis [28]. Because of the limited number of available antifungal drugs, repurposing of drugs can increase the portfolio of possible drug combinations.

Colistin is an antibiotic that targets the external membrane of gram-negative bacteria [29], but has also shown activity against *Aspergillus nidulans* and *Aspergillus niger* when combined with isavuconazole, but unfortunately not against the most common human pathogenic species *A. fumigatus* [30]. To overcome this limitation, we explored the activity of colistin in combination with amphotericin B, a wide-spectrum antifungal against *Aspergillus* species. As the combination of isavuconazole with colistin has also shown synergistic activity against *Candida auris* [31], *Candida* species including *C. auris* have also been tested in the present study with the combination of amphotericin B and colistin. Finally, to complete the portfolio of fungal infections in COVID-19 patients, the study was expanded by the inclusion of *Rhizopus* species.

## 2. Materials and Methods

### 2.1. Strains

This study included a total of 86 strains comprising of 47 *Candida* spp., 29 *Aspergillus* spp., and 10 *Rhizopus* spp., strains. *Candida* strains comprised of 10 *Candida albicans*, 15 *Candida auris*, five *Candida glabrata*, three *Candida kefyr*, five *Candida krusei*, four *Candida parapsilosis* and five *Candida tropicalis*. All *Candida* strains are clinical stains either obtained from the Westerdijk Fungal Biodiversity Institute collection (*C. auris*), or from the Department of Microbiology of the University Hospital Marburg and were identified to the species level by sequencing of the complete ITS1-5.8S-ITS2 region as described elsewhere [32]. Sequences were deposited at GenBank under the accession numbers OL351325 to OL351356. *Aspergillus* strains comprised of five *Aspergillus flavus*, 10 *Aspergillus fumigatus*, four *Aspergillus nidulans*, five *Aspergillus niger*, and five *Aspergillus terreus*. Strains were identified to the species level previously by sequencing part of the beta-tubulin and/or calmodulin genes [30]. Strains of the A. *nidulans* species complex comprised of three *Aspergillus nidulans sensu stricto* and one *Aspergillus latus*. Strains of *A. niger* species complex comprised of one *Aspergillus luchuensis*, two *Aspergillus tubingensis*, two *Aspergillus wellwitschiae*. *A. fumigatus* strains included five azole-resistant strains (four strains with TR34/L98H alterations and the other with G54W mutation). *Rhizopus* strains comprised of seven *Rhizopus arrhizus*, one *Rhizopus delemar* and two *Rhizopus microsporus*. *Rhizopus* strains that did not belong to the collection of Westerdijk Fungal Biodiversity Institute, were identified to the species level by sequencing of the complete ITS1-5.8S-ITS2 region previously [32]. Each series of experiments included the quality control reference strains *C. krusei* ATCC 6258 and *C. parapsilosis* ATCC 22019.

### 2.2. Medium Preparation

The test medium Roswell Park Memorial Institute 1640 (RPMI) medium (with L-glutamine, with pH indicator, but without bicarbonate) (Merck, Darmstadt, Germany) was prepared in double strength and contained 2% (*w*/*v*) of D-Glucose buffered with 3-(*N*-morpholino)propanesulfonic acid (Merck) at a final concentration of 0.165 mol/L. After pH adjustment, the medium was filter sterilized [33,34].

### 2.3. Drugs and Microplate Preparation

European Committee on Antimicrobial Susceptibility Testing (EUCAST) guidelines for antifungal susceptibility testing of yeasts and molds with modifications for broth microdilution checkerboard procedures were used in this study [33,34]. Nunclon^TM^ delta surface 96-wells microtiter plates for adherent cells (Thermo Fisher Scientific, Darmstadt, Germany) were used. Drugs tested in combination were amphotericin B (Merck), and colistin (Merck). Final concentrations tested ranged from 0.03 to 16 µg/mL, and from 1 to 64 µg/mL for amphotericin B and for colistin, respectively. Before the addition of the inoculum, each well contained 100 µL of double strength RMPI medium with 1% (*v*/*v*) of DMSO.

### 2.4. Inoculum Preparation and Inoculation of Microplates

All strains were subcultured twice from frozen stocks on Sabouraud dextrose agar slants supplemented with chloramphenicol and gentamycin (Bio-Rad Laboratories, Feldkirchen, Germany) at 35 °C and 95% humidity. Incubation time was 24 h for *Candida* spp. and 7 days for filamentous fungi in accordance with EUCAST recommendations for slow growing molds [33]. Suspensions were counted in a hemocytometer and adjusted to the final inoculum size of 2 × 10^5^ colony forming units (CFU)/mL in water for yeasts, and water containing 0.1% (*v*/*v*) of Tween 80 for molds, which should prevent fungal growth on surfaces of the wells [35]. After the distribution of 100 µL of the final inoculum into each well, microplates were incubated at 35 °C, with 95% humidity. Incubation time was 24 h for *Candida* and *Rhizopus* species and 48 h for *Aspergillus* species. After incubation optical densities were read spectrophotometrically at a wavelength of 530 nm using a MultiSkan FC spectrometer (Thermo Fisher Scientific). Before the reading, microplates containing yeast inocula were shaken for 2 min at 1100 rpm with a PMS-1000 Microplate Shaker (Grant Instruments, Shepreth, UK). All experiments were run in duplicate.

### 2.5. Interpretation of the Results by Fractional Inhibition Concentration Index

After subtraction of the blank plates, the optical density values from the microplates were transformed into a percentage of growth compared to the growth control. For yeast, MICs of amphotericin B were determined as the concentration that resulted in an inhibition of 90% [34], and MICs for colistin or in combination that resulted in an inhibition of 50% compared to the growth control (primary inhibition endpoint). Additionally, FICIs for the endpoints of 90% and 50% of inhibition for both drugs and in combination were calculated (additional inhibition endpoints). For molds, a 90% of inhibition endpoint for drugs alone and in combination was chosen. High off-scale MICs were converted to the next log_2_ dilution. If the lowest fractional inhibition concentration index (FICI) on the microplate was ≤0.5, or >0.5 to 4 synergy or indifference (no interaction) were assumed, respectively. If a FICI was >4.0, antagonism was concluded [36].

### 2.6. Interpretation of the Results by Response Surface Analysis

The major advantage of the Bliss independence model is its independence of MIC endpoints and MIC definitions, as it compares the effects of drugs alone, or in combination, instead of concentrations. Based on the hypothesis that drugs act independently from each other, the indifference of the combination is achieved, when the sum of the effects of the drugs alone is equal to the effect of the combination. The effect of the combination can be synergistic or antagonistic when the observed effect is better or worse compared to the expected indifferent interaction. Briefly, from the data of the microplates consisting of the percentage of growth compared to the growth control, a dose-response curve for each drug alone is generated. These dose–response curves serve to calculate a theoretical response surface of an indifferent interaction of the two drugs. This surface was then compared to the experimental surface and the synergy distribution was calculated. All calculations were performed by the Combenefit software (Windows v2.02) [37]. The synergy distribution was evaluated using three metrics: the SYN-SUM, the ANT-SUM, and the SUM-SYN-ANT. This later metric consists of the sum of synergy and antagonism observed by comparison of the two surfaces. To determine the threshold of the metric, a response surface with an indifferent interaction was determined experientially. Therefore, the combination of an antifungal with itself (amphotericin B + amphotericin B) was tested by checkerboard in triplicate. Based on the results of the experimental plates, synergy was assumed when the SUM-SYN-ANT was ≥43.8%, and antagonism was assumed when ≤−43.8%. Between –43.8 and 43.8%, indifference was concluded [38]. To determine the SUM-SYN-ANT of the different strains, the results of both runs were combined.

## 3. Results

**Figure 1:**
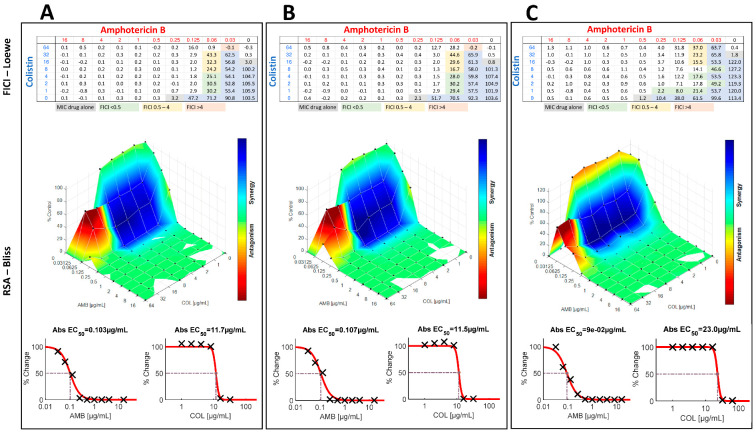
Interaction between amphotericin B (AMB) and colistin (COL) against *Candida tropicalis* isolates V2105128 (panel **A**), V2105245 (panel **B**), and V2106298 (panel **C**), showing both synergistic and antagonistic interactions depending on the concentrations. In each panel, are presented the percentage of growth and fractional inhibitory concentration index (FICI) determination (primary inhibition endpoint) based on the Loewe additivity model (top), the response-surface analysis based on the Bliss independence model (middle), and the concentration-activity response curves of the drugs alone (bottom). For the colistin concentration-activity curve against V2106298, growth at low concentrations were normalized to 100%.

**Figure 2:**
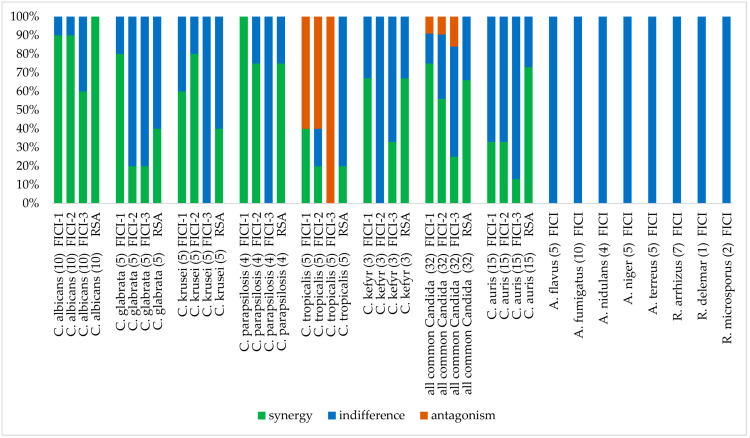
Summary of the in vitro interactions of amphotericin B with colistin against fungi responsible for invasive infections evaluated by European Committee on Antimicrobial Susceptibility Testing (EUCAST) broth microdilution checkerboard methodology and interpretation by fractional concentration index (FICI) using different inhibition endpoints and response surface analysis. FICI-1, 90% inhibition for amphotericin, 50% inhibition for colistin and in combination; FICI-2, 50% inhibition for both drugs and in combination; FICI-3, 90% inhibition for both drugs and in combination; RSA, response surface analysis.

The interactions of amphotericin B with colistin were evaluated by checkerboard against all fungal species. Interpretation of the results by FICI or by response surface analysis against strains of *Candida* spp. and *C. auris* are presented in Table 1 and Table 2, respectively. A comparison of FICI and response surface analysis for selected *C. tropicalis* strains is presented in Figure 1. The additionally calculated FICIs using 50% or 90% of inhibition are presented in Table 3 and Table 4 for *Candida* spp. and *C. auris*, respectively. Interpretation of the results by FICI of strains of *Aspergillus* spp. and *Rhizopus* spp. are presented in Table 5 and Table 6, respectively. A summary of all results is presented in Figure 2.

Using the primary inhibition endpoint, the 32 *Candida* strains (except *C. auris*) exhibited MICs for amphotericin B alone ranging from 0.25 to 0.5 μg/mL (Table 1) with a MIC50, MIC90, and geometric mean MIC of 0.25, 0.5, and 0.35 μg/mL, respectively. Amphotericin B MICs ranged from 0.25 to 0.5 µg/mL for *C. albicans*, *C. glabrata*, *C. parapsilosis*, *C. tropicalis* and *C. kefyr* and were 0.5 µg/mL for *C. krusei*. Colistin showed activity against certain species or strains of the 32 *Candida* strains (except *C. auris*) tested. MICs for colistin ranged from 16 to >64 μg/mL (128 μg/mL used as the high-off scale MIC) with a MIC50, and a geometric mean MIC of >64, and 74.48 μg/mL, respectively. The best activity of colistin was seen against *C. tropicalis* with MICs ranging from 16 to 32 µg/mL. MICs of *C. krusei* and *C. kefyr* were 64 µg/mL, except for one strain of each species (MIC of 32 µg/mL). Against *C. albicans* and *C. parapsilosis*, colistin was almost inactive, only one strain of each species had a MIC of 64 μg/mL, all other strains had higher MICs. Colistin showed no activity against *C. glabrata*, all MICs were >64 µg/mL. Between experiments, amphotericin B and colistin MICs were within +/− 1 log_2_ dilutions in 100% of the cases for all *Candida* species tested (data not shown). Interpretation of the results by fractional inhibitory concentration index showed that interaction was synergistic for 75% of the strains with FICIs ranging from 0.1328 to 0.375 with a geometric mean FICI of 0.2312. Synergy was obtained for 40, 60, 67, 80, 90% and 100% of *C. tropicalis*, *C. krusei*, *C. kefyr*, *C. glabrata*, *C. albicans* and *C. parapsilosis* strains, respectively (Figure 2). All other interactions were indifferent, except for 3 *C. tropicalis* strains. For these strains, the interaction was antagonistic. Interestingly, synergistic and antagonistic interactions were found on the same plate (Figure 1) with lowest FICIs of 0.1563 and twice 0.3125. The geometric mean FICI for all strains was 0.343, despite the inclusion of the high FICIs from the antagonistic strains.

Analysis of the checkerboard data of the 32 *Candida* strains (except *C. auris*) by the response surface approach led to similar results compared to the FICI results. Overall synergy and antagonism were obtained for 66% and none of the strains, respectively (Table 1). The SUM-SYN-ANT metric for the synergistic strains ranged from 45.14 to 87.84, with a mean of 64.74.

Synergy was obtained for 20, 40, 60, 67, 75, 100% of *C. tropicalis*, *C. glabrata*, *C. krusei*, *C. kefyr*, *C. parapsilosis* and *C. albicans*. The geometric mean SUM-SYN-ANT metric for all strains was 48.35. When comparing the results of the FICI with the response surface approach, synergy was obtained for the majority of the strains by both techniques for *C. krusei*, *C. kefyr*, *C. parapsilosis* and *C. albicans*. Interpretation of the results for *C. glabrata* was synergistic (three of five strains) by FICI and indifferent by surface analysis (three of five strains). One major difference between the interpretation techniques was that interaction against *C. tropicalis* was antagonistic (three of five strains) by FICI and indifferent by response surface analysis (four of five strains). Although the SUM-SYN-ANT metric did not reach the determined threshold, there was a trend for an antagonistic interaction by response surface analysis as shown in Figure 1.

Using the additional inhibition endpoints, globally, interactions were less synergistic and equal or more antagonistic (Table 3). Using 50% of inhibition as an endpoint, synergy was obtained for 0, 20, 20, 75, 80 and 90% of *C. kefyr, C. glabrata, C. tropicalis, C. parapsilosis, C. krusei*, *and C. albicans* strains, respectively. All other interactions were indifferent, except for 3 *C. tropicalis* strains, for which interactions were antagonistic. Using 90% of inhibition as an endpoint, synergy was obtained for 0, 0, 0, 20, 33% and 60% of *C. krusei*, *C. parapsilosis*, *C. tropicalis*, *C. glabrata*, *C. kefyr* and *C. albicans* strains, respectively. All other interactions were indifferent, except for *C. tropicalis*, for which all tested strains exhibited antagonism (Figure 2).

Using the primary inhibition endpoint, the 15 *C. auris* strains exhibited slightly higher MICs for amphotericin B alone than the other *Candida* spp. ranging from 0.5 to 1 μg/mL (Table 2) with a MIC50, and a geometric mean MIC of 1, and 0.83 μg/mL, respectively. Colistin alone showed no activity against *C. auris*, all MICs were >64 µg/mL. Between experiments, amphotericin B and colistin MICs were within +/− 1 log_2_ dilutions in 100% of the cases for all strains tested (data not shown). Interpretation of the results by fractional inhibitory concentration index led to synergistic interactions for 33% of the strains with FICIs ranging from 0.1563 to 0.375 with a geometric mean FICI of 0.2378. The geometric mean FICI for all strains was 0.3943. The geometric mean MIC for colistin in combination with the synergistic isolates was 5.28, and 1.7 µg/mL for all strains.

Response surface analysis for the 15 *C. auris* strains led to synergistic interactions for 73% of the strains (Table 2). The SUM-SYN-ANT metric for the synergistic strains ranged from 47.90 to 84.31, with a geometric mean of 56.38. All other interactions were indifferent. The geometric mean SUM-SYN-ANT metric for all strains was 47.46. When comparing the results of the FICI with the response surface approach, synergy was more frequently obtained (73 vs. 33%) (Figure 2).

Using the additional inhibitions endpoints, synergy was equally or less frequently seen, compared to the primary inhibition endpoint. Combination exhibited synergy for 33 or 13% of the strains using the 50 or 90% of inhibition endpoint, respectively.

The 29 *Aspergillus* strains exhibited MICs for amphotericin B alone ranging from 0.5 to 4 μg/mL (Table 5) with a MIC50, MIC90, and geometric mean MIC of 2, 4, and 2 μg/mL, respectively. Amphotericin B MICs ranged from 0.5 to 1 µg/mL for *A. niger*, from 2 to 4 μg/mL for *A. flavus*, *A. nidulans* and *A. terreus*, and were 2 µg/mL for *A. fumigatus*. Colistin alone showed no activity against *Aspergillus* species, all MICs were >64 µg/mL. Between experiments, amphotericin B and colistin MICs were within +/− 1 log_2_ dilutions in 100% of the cases for all *Aspergillus* species tested (data not shown). Interpretation of the results by fractional inhibitory concentration index led to indifferent interactions for all the strains tested (Figure 2).

The 10 *Rhizopus* strains exhibited MICs for amphotericin B alone ranging from 0.5 to 1 μg/mL (Table 6) with a MIC50, MIC90, and geometric mean MIC of 0.5, 1, and 0.57 μg/mL, respectively. Amphotericin B MICs for *R. arrhizus* and *R. delemar* were 0.5 and were 1 µg/mL for *R. microsporus*. Colistin alone showed activity against *Rhizopus* species with MICs ranging from 16 to 32 µg/mL with a geometric mean MIC of 18.38 µg/mL. Between experiments, amphotericin B and colistin MICs were within +/− 1 log_2_ dilutions in 100% of the cases for all *Rhizopus* species tested (data not shown). Interpretation of the results by fractional inhibitory concentration index let to indifferent interactions for all the strains tested (Figure 2).

## 4. Discussion

Colistin is an antibiotic drug of last resort with good penetration of the lungs used for the treatment of pulmonary infections due to multidrug-resistant gram-negative bacteria, such as *Pseudomonas aeruginosa*, *Klebsiella pneumoniae*, or *Acinetobacter baumanii* [39]. Its bactericidal activity is evoked by its ability to target the external membrane, leading to membrane alteration and resulting in increased membrane permeability [40]. Apart from its activity against gram-negative bacteria, cytoplasmic membrane damage has also been demonstrated in *C. albicans* and *R. arrhizus* [41,42]. We previously showed in vitro synergy of colistin in combination with isavuconazole for *A. nidulans*, *A. niger* and *C. auris* [30,31], which makes the antibiotic an interesting partner to explore combinations with other antifungals. Therefore, in this study amphotericin B was tested in vitro in combination with colistin against fungi responsible for invasive infections.

Amphotericin B MICs were in the same ranges as previously reported for *Rhizopus* species [43], *C. auris* [44], and the different *Candida* species [45,46]. For *Aspergillus* species, amphotericin B MICs were in the same range for *A. flavus*, *A. nidulans*, *A. niger* and *A. terreus*, but not for *A. fumigatus* [46]. In this study, all *A. fumigatus* MICs were 2 µg/mL in both runs (data not shown). According to the newest EUCAST breakpoint definition for *A. fumigatus* from 2020, after the elimination of the status intermediate susceptibility, a MIC of 2 µg/mL would identify an amphotericin B resistant isolate, while in the old definition a MIC of 2 µg/mL would have identified an isolate with an intermediate susceptibility [47]. As it has been shown that spectrophotometric reading is a good alternative for visual reading [48], using 90% or 95% of inhibition as an endpoint compared to the growth control [49], quality controls were within the target range for amphotericin B (Table 1), and that it is unlikely all tested *A. fumigatus* strains are resistant to amphotericin B, it remains unclear how the interpret the MICs of 2 µg/mL of these isolates.

MICs of colistin alone determined by EUCAST methodology for *Aspergillus* species and *C. auris* were the same as previously reported [30,31,50]. Colistin MICs for *Rhizopus* species by EUCAST methodology have not been determined before, but CLSI methodology MICs were in the same range [41]. Colistin combination MICs for common *Candida* species ranged from 1 to 2 µg/mL (except for *C. tropicalis*), which would be in the range of peak serum levels reported in patients with cystic fibrosis [51], and critically ill patients [52]. The geometric mean MIC of colistin in combination with the synergistic *C. auris* isolates was slightly higher (5.28 µg/mL), but was still in the range of the achievable serum levels.

In this study, we analyzed the checkerboard data of *Candida* species by interpretation of the results by FICI, or response surface analysis. One of the disadvantages of the FICI technique is its dependence on the MIC endpoints. Another problem is the definition of the endpoint itself, as 50% or 90% of growth inhibition compared to the growth control can be used, using either can lead to completely different conclusions [53,54]. For combination studies, no standardized methods exist, especially if one of the partners belongs to another drug class (in our case antifungal and antibiotic). In this study, we have chosen 90% of inhibition for amphotericin B for *Candida* species as recommended by EUCAST [34], and 50% for colistin and in combination. EUCAST recommends using 50% of inhibition for all other antifungals except amphotericin B, but of course, colistin is not comparable to other antifungals. To overcome these limitations of the FICI approach, we additionally interpreted the checkerboard results by response surfaces analysis. The great advantage of this approach is its independence of MIC endpoints and definitions, as it compares the effects of drugs alone, or in combination, instead of concentrations [38].

The use of different endpoints for drugs alone and for the combination has already been reported in previous studies [55,56]. The influence of the reading endpoint has also been evaluated in previous studies [57], and showed that using a 90% inhibition endpoint led to less detection of synergy. In the present study, the use of 90% inhibition for amphotericin B and 50% for colistin and combination for the FICI calculation showed the best agreement with the response surface analysis results (75% synergy by FICI and 66% by response surface analysis for *Candida* spp.) and has, therefore, been chosen as primary inhibition endpoint. The additionally evaluated inhibition endpoints of 50 or 90% of inhibition for both drugs and in combination globally exhibited less synergistic and equal or more antagonistic interactions. Synergy was detected for 56 or 25% of the tested strains, and antagonism for three of five, or five of five *C. tropicalis* strains, when 50 or 90% of inhibition was used as an endpoint, respectively. The different results obtained with the different endpoints or methods (FICI vs. response surface analysis) could be explained by the fact that using a 50% inhibition endpoint, or response surface analysis may capture interactions at the sub-MIC level that are not captured with the FICI when using a 90% inhibition endpoint.

Apart of the two studies from our laboratories mentioned above [30,31], synergy of colistin in combinations with antifungals has been reported for yeasts [42,50,58,59,60,61], and filamentous fungi [42,60], but indifference [42,50,61,62], and antagonism [62,63] have also been reported. In this study, using the primary inhibition endpoint, we found synergy of the combination of amphotericin B and colistin for common *Candida* species except for *C. tropicalis* by both approaches (75% for FICI and 66% for response surface analysis). Two studies showed synergy for the combination of amphotericin B with colistin, but each study tested only one *C. albicans* strain [42,61]. These results are in accordance with our study. As previously suggested [42], the membrane damage probably induced by colistin could be enhanced by the known permeabilization of the membrane by amphotericin B, and could, therefore, explain the synergistic effect observed when these two drugs are combined. Another study evaluated the combination of liposomal amphotericin B and colistin against five *Candida* strains belonging to different species. Unfortunately, only amphotericin B combination MICs were shown, and not colistin combinations MICs, which makes an interpretation of the results impossible [60]. Against *C. albicans*, the combination of colistin with caspofungin or fluconazole was synergistic in vitro and in vivo in *Galleria mellonella* [58,64]. Echinocandins were also found synergistic in combination with colistin, but the number of strains tested was limited [61].

Interaction of the combination against *C. tropicalis* was antagonistic for three isolates and synergistic for two isolates by FICI. Interestingly, synergistic and antagonistic FICIs were simultaneously present on checkboard microplates of all 3 antagonistic strains (Figure 1). By definition, if there is at least one FICI ≥ 4, the highest FICI is retained [38]. It is unclear if it has been considered that synergistic and antagonistic interactions can be present on the same microplate when this definition was set-up. Interpretation by response surface analysis showed indifferent interactions for four strains and synergistic for the other. The ANT-SUM of the five *C. tropicalis* isolates ranges from −8.56 to −16.84, but does not meet the definition of antagonism of −43.8; and certainly not if the SYN-SUM is added. Which interaction of the two approaches represents the reality remains unknown. To answer this question, animal experiments are required.

For *C. auris* response surface analysis showed 73% of synergy for the combination, while by FICI the combination exhibited synergy for only 33% of the tested strains, using the primary inhibition endpoint. While the geometric mean FICI of all isolates was quite high (0.39), the geometric mean SUM-SYN-ANT was low (47.46). These numbers underline that the synergy of the combination against *C. auris* is only weak. This could explain the discrepancy between the two approaches, maybe the FICI is not sensitive enough to demonstrate the weak synergy of the combination against *C. auris*. Two other studies evaluated colistin in combination with antifungals against *C. auris*. In the first study, the combination of isavuconazole with colistin was synergistic by FICI and response surface analysis, but an agar diffusion assay was not sensitive enough to demonstrate synergy, despite a MIC reduction for the combination of all tested strains compared to the drugs alone [31]. In the second study combination of caspofungin or micafungin with colistin showed synergistic and indifferent interactions, respectively [50].

We found indifferent interactions for the combination against all strains of the tested *Aspergillus* species using 90% of inhibition for both drugs alone and in combination compared to the growth control. Additionally, sub-MIC evaluation, using an endpoint of 50% of inhibition, showed no significantly different interactions (data not shown). One other study evaluated the combination of liposomal amphotericin B and colistin against three *A. fumigatus* strains. MICs of amphotericin B in combination were significantly reduced, but it is unclear if combination MICs of colistin were significantly reduced [60]. A combination of colistin with isavuconazole was tested against different *Aspergillus* species, the synergy of the combination was demonstrated for *A. nidulans* and *A. niger*, but agar diffusion assays were not sensitive enough to confirm the synergy. The combination was synergistic for 40% of the tested *A. niger* strains and indifferent for the rest of the tested *A. niger* strains, and for all *A. nidulans* strains tested [30].

One *Lichtheimia corymbifera* isolate was tested using colistin in combination with amphotericin B or itraconazole. Both combinations exhibited synergy [42]. However, in this study combination of amphotericin B with colistin exhibited only indifference against all *Rhizopus* species strains tested. As for *Aspergillus* species, sub-MIC evaluation using an endpoint of 50% of inhibition showed no significantly different interactions (data not shown).

It should be noted that combining two nephrotoxic drugs, such as amphotericin B and colistin may be problematic in patients. Nevertheless, for difficult to treat fungal infections it could be discussed if the benefit of the combination may outweigh the potential toxicity. More importantly, this study is a proof of concept and suggests that drugs active on the bacterial membrane can be synergistic when used in combination with antifungals, and this could stimulate the research and development of new drugs with less nephrotoxicity.

In summary, colistin enhances the in vitro activity of amphotericin B against *Candida* species, except for *C. tropicalis* for which the results differed between the interpretation models. Against *Aspergillus* and *Rhizopus* species the combination was indifferent for all strains tested. The results of the experiments obtained for the *Candida* species warrant further in vivo experiments.

## Figures and Tables

**Table 1:** Interaction of amphotericin B with colistin against common *Candida* spp. by checkerboard and interpretation by fractional inhibitory concentration index using 90% of inhibition for amphotericin B and 50% of inhibition for colistin and in combination, and by response surface analysis.

Species	Collection Number	Checkerboard MICs (µg/mL)					Response Surface Analysis	
		AMB	COL	AMB/COL	FICI	INTPN	ΣSYN-ANT (ΣSYN; ΣANT)	INTPN
*C. albicans*	V2105126	0.25	>68	0.06/1	0.2578	SYN	81.36 (81.48; −0.12)	SYN
*C. albicans*	N2101578	0.5	>68	0.125/1	0.2578	SYN	79.68 (80.13; −0.45)	SYN
*C. albicans*	V2105568	0.25	>68	0.06/2	0.2656	SYN	72.40 (72.73; −0.33)	SYN
*C. albicans*	N2101577	0.25	>68	0.125/1	0.5078	IND	62.97 (63.75; −0.78)	SYN
*C. albicans*	V2105825iso3	0.25	>68	0.03/1	0.1328	SYN	76.46 (76.73; −0.27)	SYN
*C. albicans*	ATCC 14053	0.25	>68	0.06/1	0.2578	SYN	80.98 (82.44; −1.46)	SYN
*C. albicans*	V2105529	0.25	>68	0.03/1	0.1328	SYN	87.84 (87.89; −0.05)	SYN
*C. albicans*	V2106139	0.25	>68	0.06/1	0.2578	SYN	80.84 (81.29: −0.45)	SYN
*C. albicans*	V2106041	0.25	64	0.06/1	0.2656	SYN	79.58 (82.62; −3.04)	SYN
*C. albicans*	V2106305	0.25	>68	0.03/2	0.1406	SYN	70.02 (70.43; −0.41)	SYN
*C. glabrata*	V2105272	0.5	>68	0.25/1	0.5078	IND	52.39 (53.11; −0.72)	SYN
*C. glabrata*	V2105282	0.5	>68	0.125/2	0.2656	SYN	10.67 (11.28; −0.61)	IND
*C. glabrata*	N2101711	0.5	>68	0.125/2	0.2656	SYN	21.72 (23.48: −1.76)	IND
*C. glabrata*	V2105636	0.5	>68	0.125/2	0.2656	SYN	32.39 (33.08; −0,69)	IND
*C. glabrata*	DSM 70614	0.25	>68	0.06/1	0.2578	SYN	66.18 (67.17; −0.99)	SYN
*C. krusei*	V2105825iso4	0.5	64	0.125/2	0.2813	SYN	82.38 (83.33; −0.95)	SYN
*C. krusei*	V2105866	0.5	64	0.125/1	0.2656	SYN	49.18 (53.96; −4.78)	SYN
*C. krusei*	V2106177	0.5	64	0.25/1	0.5156	IND	43.68 (47.79; −4.11)	IND
*C. krusei*	V2105920	0.5	64	0.25/1	0.5156	IND	42.66 (44.51; −1.85)	IND
*C. krusei*	ATCC 6258	0.5	32	0.125/4	0.375	SYN	45.14 (47.64; −2.50)	SYN
*C. parapsilosis*	V2105056	0.25	>68	0.06/1	0.2578	SYN	45.94 (45.96; −0.02	SYN
*C. parapsilosis*	V2105223	0.25	>68	0.06/1	0.2578	SYN	48.52 (49.02; −0.50)	SYN
*C. parapsilosis*	B2107379	0.5	>68	0.06/2	0.1406	SYN	39.86 (40.36; −0.50)	IND
*C. parapsilosis*	ATCC 22019	0.5	64	0.06/1	0.1406	SYN	69.92 (70.41; −0.49)	SYN
*C. tropicalis*	V2105128	0.25	16	0.03/64	4.125	ANT	28.11 (43.14, −15.03)	IND
*C. tropicalis*	V2105245	0.25	16	0.03/64	4.125	ANT	21.56 (38.40; −16.84)	IND
*C. tropicalis*	V2105598	0.25	16	0.03/1	0.1875	SYN	17.29 (25.85; −8.56)	IND
*C. tropicalis*	B1907975	0.25	32	0.06/1	0.2813	SYN	36.13 (44.84; −8.71)	IND
*C. tropicalis*	V2106298	0.5	32	0.03/>68	4.125	ANT	55.85 (66.76; −10.91)	SYN
*C. kefyr*	V2105566	0.25	64	0.125/1	0.5156	IND	54.03 (63.85; −9,82)	SYN
*C. kefyr*	V2106126	0.5	64	0.125/1	0.2656	SYN	52.41 (54.37; −1.96)	SYN
*C. kefyr*	N2101899	0.5	32	0.125/1	0.2813	SYN	33.27 (36.56; −3.29)	IND

FICI, fractional inhibitory concentration index; INTPN, interpretation; SYN, synergy; IND, no interaction; ANT, antagonism; AMB, amphotericin B; COL, colistin; ATCC, American Type Culture Collection; DSM, Deutsche Sammlung von Mikroorganismen und Zellkulturen.

**Table 2:** Interaction of amphotericin B with colistin against *Candida auris* by checkerboard and interpretation by fractional inhibitory concentration index using 90% of inhibition for amphotericin B and 50% of inhibition for colistin and in combination, and response surface analysis.

Species	Collection Number	Checkerboard MICs (µg/mL)					Response Surface Analysis	
		AMB	COL	AMB/COL	FICI	INTPN	ΣSYN-ANT (ΣSYN; ΣANT)	INTPN
*C. auris*	CBS 10913	0.5	>68	0.06/4	0.1563	SYN	84.31 (84.55; −0.24)	SYN
*C. auris*	CBS 12372	0.5	>68	0.125/8	0.3125	SYN	32.54 (34.01; −1.47)	IND
*C. auris*	CBS 12373	0.5	>68	0.125/2	0.2656	SYN	69.35 (70.74; −1.39)	SYN
*C. auris*	CBS 12766	1	>68	0.5/1	0.5078	IND	26.82 (28.55; −1.73)	IND
*C. auris*	CBS 12767	1	>68	0.5/1	0.5078	IND	40.81 (45.53; −4.72)	IND
*C. auris*	CBS 12768	1	>68	0.5/1	0.5078	IND	47.90 (48.96; −1.06)	SYN
*C. auris*	CBS 12769	1	>68	0.5/1	0.5078	IND	21.41 (21.63; −0.22)	IND
*C. auris*	CBS 12770	1	>68	0.25/16	0.375	SYN	56.41(56.67; −0.26)	SYN
*C. auris*	CBS 12771	1	>68	0.5/1	0.5078	IND	51.08 (51.37; −0.29)	SYN
*C. auris*	CBS 12772	1	>68	0.5/1	0.5078	IND	54.38 (54.50; −0.12)	SYN
*C. auris*	CBS 12773	1	>68	0.5/1	0.5078	IND	56.26 (56.28; −0.02)	SYN
*C. auris*	CBS 12774	1	>68	0.5/1	0.5078	IND	53.74 (53.76; −0.02)	SYN
*C. auris*	CBS 12775	1	>68	0.5/1	0.5078	IND	51.09 (51.14; −0.05)	SYN
*C. auris*	CBS 12776	1	>68	0.5/1	0.5078	IND	44.93 (46.24; −1.31)	SYN
*C. auris*	CBS 12777	0.5	>68	0.06/4	0.1563	SYN	60.03 (60.27; −0.24)	SYN

FICI, fractional inhibitory concentration index; INTPN, interpretation; SYN, synergy; IND, no interaction; AMB, amphotericin B; COL, colistin; CBS, Westerdijk Fungal Biodiversity Institute.

**Table 3:** Interaction of amphotericin B with colistin against common *Candida* spp. by checkerboard and interpretation by fractional inhibitory concentration index using 50%, or 90% of inhibition for both drugs and in combination.

Species	Collection Number	Inhibition Endpoint			
		50%		90%	
		FICI	INTPN	FICI	INTPN
*C. albicans*	V2105126	0.2578	SYN	0.3125	SYN
*C. albicans*	N2101578	0.5078	IND	0.3125	SYN
*C. albicans*	V2105568	0.2656	SYN	0.5078	IND
*C. albicans*	N2101577	0.3125	SYN	0.5078	IND
*C. albicans*	V2105825iso3	0.2578	SYN	0.2578	SYN
*C. albicans*	ATCC 14053	0.2578	SYN	0.5078	IND
*C. albicans*	V2105529	0.2578	SYN	0.2578	SYN
*C. albicans*	V2106139	0.2578	SYN	0.5078	IND
*C. albicans*	V2106041	0.375	SYN	0.2656	SYN
*C. albicans*	V2106305	0.2656	SYN	0.2813	SYN
*C. glabrata*	V2105272	0.5078	IND	0.5078	IND
*C. glabrata*	V2105282	0.5156	IND	0.5078	IND
*C. glabrata*	N2101711	0.5156	IND	0.5078	IND
*C. glabrata*	V2105636	0.5156	IND	0.5078	IND
*C. glabrata*	DSM 70614	0.2578	SYN	0.3125	SYN
*C. krusei*	V2105825iso4	0.2813	SYN	0.5313	IND
*C. krusei*	V2105866	0.5156	IND	0.5156	IND
*C. krusei*	V2106177	0.3125	SYN	0.5156	IND
*C. krusei*	V2105920	0.2813	SYN	0.5313	IND
*C. krusei*	ATCC 6258	0.375	SYN	0.5313	IND
*C. parapsilosis*	V2105056	0.5078	IND	0.5156	IND
*C. parapsilosis*	V2105223	0.2578	SYN	0.5313	IND
*C. parapsilosis*	B2107379	0.2656	SYN	0.5078	IND
*C. parapsilosis*	ATCC 22019	0.2656	SYN	0.5078	IND
*C. tropicalis*	V2105128	4.25	ANT	8.5	ANT
*C. tropicalis*	V2105245	4.125	ANT	8.5	ANT
*C. tropicalis*	V2105598	0.3125	SYN	8.5	ANT
*C. tropicalis*	B1907975	0.5313	IND	4.5	ANT
*C. tropicalis*	V2106298	4.5	ANT	4.25	ANT
*C. kefyr*	V2105566	0.5156	IND	0.5156	IND
*C. kefyr*	V2106126	0.5156	IND	0.375	SYN
*C. kefyr*	N2101899	0.5313	IND	0.5313	IND

FICI, fractional inhibitory concentration index; INTPN, interpretation; SYN, synergy; IND, no interaction; ANT, antagonism; AMB, amphotericin B; COL, colistin; ATCC, American Type Culture Collection; DSM, Deutsche Sammlung von Mikroorganismen und Zellkulturen.

**Table 4:** Interaction of amphotericin B with colistin against *C. auris* by checkerboard and interpretation by fractional inhibitory concentration index using 50%, or 90% of inhibition for both drugs and in combination.

Species	Collection Number	Inhibition Endpoint			
		50%		90%	
		FICI	INTPN	FICI	INTPN
*C. auris*	CBS 10913	0.2813	SYN	0.2656	SYN
*C. auris*	CBS 12372	0.3125	SYN	0.5078	IND
*C. auris*	CBS 12373	0.2656	SYN	0.5078	IND
*C. auris*	CBS 12766	0.5078	IND	0.5313	IND
*C. auris*	CBS 12767	0.5078	IND	0.5156	IND
*C. auris*	CBS 12768	0.5078	IND	0.5078	IND
*C. auris*	CBS 12769	0.5078	IND	0.5078	IND
*C. auris*	CBS 12770	0.375	SYN	0.5078	IND
*C. auris*	CBS 12771	0.5078	IND	0.5156	IND
*C. auris*	CBS 12772	0.5078	IND	0.5078	IND
*C. auris*	CBS 12773	0.5078	IND	0.5078	IND
*C. auris*	CBS 12774	0.5078	IND	0.5156	IND
*C. auris*	CBS 12775	0.5078	IND	0.5313	IND
*C. auris*	CBS 12776	0.5078	IND	0.5156	IND
*C. auris*	CBS 12777	0.2813	SYN	0.2656	SYN

FICI, fractional inhibitory concentration index; INTPN, interpretation; SYN, synergy; IND, no interaction; AMB, amphotericin B; COL, colistin; CBS, Westerdijk Fungal Biodiversity Institute.

**Table 5:** Interaction of amphotericin B with colistin against *Aspergillus* spp. by checkerboard and interpretation by fractional inhibitory concentration index using 90% of inhibition for both drugs and in combination.

Species	Collection Number	MIC (µg/mL)				
		AMB	COL	AMB/COL	FICI	INTPN
*A. flavus*	HEGP-6097	2	>68	2/1	1.0078	IND
*A. flavus*	HEGP-5899	2	>68	2/1	1.0078	IND
*A. flavus*	HEGP-4536	4	>68	4/4	1.0313	IND
*A. flavus*	HEGP-4251	2	>68	2/2	1.0156	IND
*A. flavus*	HEGP-4114	4	>68	4/1	1.0078	IND
*A. fumigatus*	HEGP-5780	2	>68	1/8	0.5625	IND
*A. fumigatus*	HEGP-4020	2	>68	1/4	0.5313	IND
*A. fumigatus*	HEGP-4083	2	>68	1/4	0.5313	IND
*A. fumigatus*	HEGP-2659	2	>68	1/16	0.625	IND
*A. fumigatus*	HEGP-2664	2	>68	2/1	1.0078	IND
*A. fumigatus*	HEGP-R117	2	>68	1/8	0.5625	IND
*A. fumigatus*	HEGP-R279	2	>68	1/2	0.5156	IND
*A. fumigatus*	HEGP-R285	2	>68	1/4	0.5313	IND
*A. fumigatus*	HEGP-R290	2	>68	1/16	0.625	IND
*A. fumigatus*	HEGP-R291	2	>68	1/4	0.5313	IND
*A. nidulans*	HEGP-5711	4	>68	0.5/64	0.625	IND
*A. nidulans*	HEGP-6169	2	>68	2/1	1.0078	IND
*A. nidulans*	HEGP-5521	4	>68	4/1	1.0078	IND
*A. nidulans*	HEGP-5329	2	>68	2/1	1.0078	IND
*A. niger*	HEGP-6071	1	>68	1/1	1.0078	IND
*A. niger*	HEGP-6217	1	>68	1/1	1.0078	IND
*A. niger*	HEGP-6475	0.5	>68	0.5/1	1.0078	IND
*A. niger*	HEGP-6562	0.5	>68	0.5/1	1.0078	IND
*A. niger*	HEGP-6917	0.5	>68	0.5/1	1.0078	IND
*A. terreus*	HEGP-6625	4	>68	4/1	1.0078	IND
*A. terreus*	HEGP-6055	4	>68	4/1	1.0078	IND
*A. terreus*	HEGP-5599	4	>68	4/1	1.0078	IND
*A. terreus*	HEGP-5169	2	>68	2/1	1.0078	IND
*A. terreus*	HEGP-6398	4	>68	4/1	1.0078	IND

FICI, fractional inhibitory concentration index; INTPN, interpretation; IND, no interaction; AMB, amphotericin B; COL, colistin; HEGP, Hôpital Européen Georges-Pompidou. Within the strains of *A. nidulans* species complex there were 3 *A. nidulans sensu stricto* and 1 *A. latus*. Within the strains of *A. niger* species complex there were 1 *A. luchuensis*, 2 *A. tubingensis*, 2 *A. wellwitschiae*.

**Table 6:** Interaction of amphotericin B with colistin against *Rhizopus* spp. by checkerboard and interpretation by fractional inhibitory concentration index using 90% of inhibition for both drugs and in combination.

Species	Collection Number	Checkerboard MICs (µg/mL)				
		AMB	COL	AMB/COL	FICI	INTPN
*R. arrhizus*	CBS 120809	0.5	16	0.25/8	1	IND
*R. arrhizus*	IP 4.77	0.5	16	0.25/4	0.75	IND
*R. arrhizus*	CBS 112.07	0.5	16	0.5/1	1.0625	IND
*R. arrhizus*	CBS 120590	0.5	16	0.25/8	1	IND
*R. arrhizus*	CBS 120591	0.5	16	0.25/4	0.75	IND
*R. arrhizus*	CBS 120808	0.5	32	0.03/16	0.5625	IND
*R. arrhizus*	IP 1443.75	0.5	16	0.25/4	0.75	IND
*R. delemar*	CBS 120593	0.5	32	0.25/8	0.75	IND
*R. microsporus*	CBS 120955	1	16	0.5/4	0.75	IND
*R. microsporus*	IP 676.72	1	16	0.5/1	0.5625	IND

FICI, fractional inhibitory concentration index; INTPN, interpretation; IP, Institut Pasteur; SYN, synergy; IND, no interaction; AMB, amphotericin B; COL, colistin; CBS, Westerdijk Fungal Biodiversity Institute.

## Data Availability

Not applicable.
